# *In Vivo* Assessment of Peripheral and Spinal Neuronal Activity in the PSNL Model: Insights into Neuropathic Pain Mechanisms

**DOI:** 10.3390/ijms27010124

**Published:** 2025-12-22

**Authors:** Daisuke Uta, Takuya Yamane, Sosuke Yoneda, Erika Kasai, Toshiaki Kume

**Affiliations:** 1 Department of Applied Pharmacology, Faculty of Pharmaceutical Sciences, University of Toyama, Toyama 930-0194, Toyama, Japan; tkume@pha.u-toyama.ac.jp; 2 Department of Applied Pharmacology, Graduate School of Medicine and Pharmaceutical Sciences, University of Toyama, Toyama 930-0194, Toyama, Japan; d2368301@ems.u-toyama.ac.jp; 3 Neuroscience, Drug Discovery & Disease Research Laboratory, Shionogi & Co., Ltd., Toyonaka 561-0825, Osaka, Japan; sosuke.yoneda@shionogi.co.jp (S.Y.); erika.kasai@shionogi.co.jp (E.K.)

**Keywords:** neuropathic pain, *in vivo* extracellular recordings, spinal cord, dorsal root ganglion, partial sciatic nerve ligation

## Abstract

Neuropathic pain represents a critical challenge in medical research and clinical practice. Enhanced peripheral nerve activity and spinal dorsal horn neuronal firing are thought to contribute to the nociceptive hypersensitivities that are observed in chronic pain conditions, including those modeled by partial sciatic nerve ligation (PSNL). However, the detailed *in vivo* neuronal response dynamics and underlying mechanisms in the PSNL model remain to be fully clarified. To better understand these mechanisms, we evaluated dorsal root ganglion (DRG) and spinal dorsal horn neuronal activity in the PSNL model using *in vivo* approaches. Von Frey testing revealed sustained mechanical allodynia in PSNL animals; withdrawal thresholds were significantly reduced up to day 14 post-surgery. Immunohistochemistry revealed a stimulation-dependent increase in phosphorylated extracellular signal-regulated kinase (pERK)-positive neurons in the DRG, thereby indicating heightened peripheral nerve activity. Additionally, electrophysiological recordings demonstrated the enhanced firing of spinal dorsal horn neurons in response to the same stimuli. Notably, DRG pERK expression changes correlated with spinal neuronal firing frequency. Together, these findings suggest that peripheral nerve activity drives spinal neuronal sensitization, thus elucidating both pain mechanisms in the PSNL model and activity-dependent signaling in neuropathic pain.

## 1. Introduction

Chronic pain—typically defined as pain persisting for more than 3 months—is an important global health issue that affects over 30% of individuals worldwide [1,2]. It negatively affects both physical and mental well-being, disrupts multiple aspects of daily life, and greatly reduces quality of life. Among the various causes of chronic pain, neuropathic pain has garnered particular attention because of its complex pathophysiology and limited treatment options [1].

Neuropathic pain is defined as pain that is caused by a lesion or disease of the somatosensory nervous system; it can result from conditions such as diabetic neuropathy, postherpetic neuralgia, and spinal cord injury [3]. A key feature of neuropathic pain is the sensitization of the nervous system following nerve injury. This sensitization leads to exaggerated responses to normally non-painful stimuli (allodynia) and increased pain from normally painful stimuli (hyperalgesia) [3]. Such abnormal sensitivity occurs both in the peripheral and central nervous systems, thus highlighting the need for comprehensive investigations into both domains when developing effective therapeutic strategies [4]. Peripheral sensory neurons have cell bodies that are located in the dorsal root ganglia (DRG) and extend axons to both the spinal cord and peripheral tissue [5]. These neurons detect external stimuli—such as mechanical, thermal, or cold stimuli—through specialized receptors (including chemical receptors and mechanoreceptors) at their peripheral terminals [6]. Voltage-gated sodium, potassium, and calcium channels as well as transient receptor potential channels regulate membrane excitability and enable the transmission of sensory information to the spinal cord, where it is then relayed to higher brain centers and ultimately perceived as pain [7].

Using both molecular and electrophysiological techniques, extensive research has been conducted to investigate alterations in neuronal activity within peripheral and spinal neurons in models of neuropathic pain. In the peripheral nervous system, studies have examined spontaneous activity in isolated and cultured DRG neurons *in vitro*, as well as *in vivo* electrophysiological recordings from the sciatic nerve and dorsal roots [8,9,10,11,12,13,14]. Other *in vivo* approaches include calcium imaging of DRG neurons and the immunostaining of activity-dependent markers [15,16]. These studies suggest the presence of peripheral hyperactivity in neuropathic pain states. Similarly, many studies have focused on spinal cord neurons to explore central sensitization. These include *in vitro* or ex vivo electrophysiological recordings from spinal cord slices, *in vivo* assessments of spontaneous firing and evoked responses, the immunohistochemical detection of activity markers (e.g., c-Fos and phosphorylated extracellular signal-regulated kinase [pERK]), and *in vivo* calcium imaging [17,18,19,20,21,22]. Together, findings from these studies indicate that spinal neurons also become hyperresponsive following nerve injury.

Most existing studies have evaluated peripheral and spinal neuronal activity independently, often using separate experimental paradigms. This fragmented approach has limited our ability to achieve an integrated understanding of neuropathic pain mechanisms. Consequently, despite extensive research efforts, the development of effective pharmacological treatments has been largely unsuccessful. To overcome these limitations, a unified, system-level analysis that concurrently examines peripheral and central nervous system activity is essential. The partial sciatic nerve ligation (PSNL) model is one of the most commonly used and well-established models for studying neuropathic pain [23]. In the present study, we aimed to address the current knowledge gap by systematically assessing both stimulus-evoked and spontaneous neuronal activity in the DRG and spinal dorsal horn of PSNL model rats using *in vivo* calcium imaging and electrophysiological techniques. Our goal was to gain deeper insights into the mechanisms underlying neuropathic pain and to contribute to the development of more effective therapeutic strategies.

## 2. Results

### 2.1. Pain Behavior

**Figure 1:**
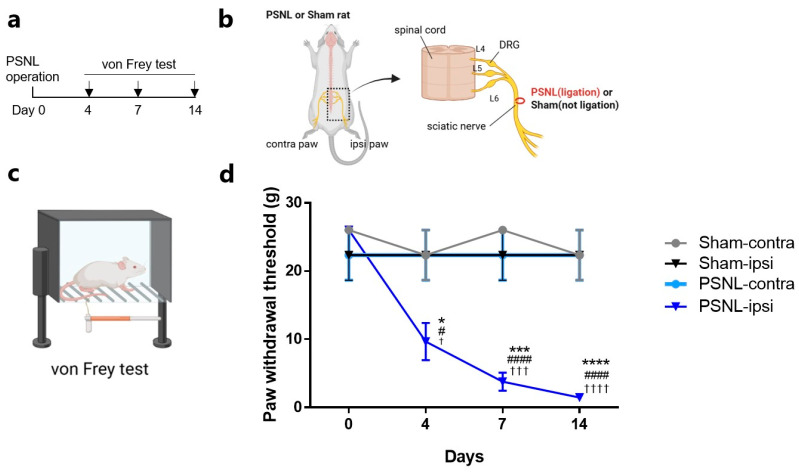
Description of the partial sciatic nerve ligation (PSNL) model and pain behavior assessment using the von Frey test. (**a**) Timeline of the model development process and the schedule for von Frey assessments. (**b**) Schematic diagram highlighting the ligation site in the PSNL model. (**c**) Illustration demonstrating the procedure of the von Frey test. (**d**) Time-course results of the von Frey test in Sham and PSNL model rats. Sham-ipsi overlapped with PSNL-contra at all time points. Statistical analysis was performed using two-way analysis of variance with post hoc Dunnett test. Data are shown as the mean ± standard error. (**c**) *n* = 3. * *p* < 0.05, *** *p* < 0.001, **** *p* < 0.0001 vs. Sham-ipsilateral. ^#^ *p* < 0.05, ^####^ *p* < 0.0001 vs. Sham-contralateral. ^†^ *p* < 0.05, ^†††^ *p* < 0.001, ^††††^ *p* < 0.0001 vs. PSNL-contralateral. Created with BioRender.com (accessed on 18 December 2025).

Pain behavior associated with the PSNL model was evaluated using the von Frey method (see Materials & Methods). Tests were performed on day 0 (prior to PSNL model establishment) and on days 4, 7, and 14 post-surgeries (Figure 1a). The PSNL model was induced by the partial ligation of the sciatic nerve, which contains the peripheral axons of sensory neurons (Figure 1b). In the von Frey test, the paw of each rat is stimulated with von Frey filaments, and withdrawal behaviors are observed to assess the pain response to mechanical stimuli (Figure 1c). There were significant reductions in withdrawal thresholds in the ipsilateral paws of PSNL model rats. These decreased thresholds were consistent across days 4, 7, and 14, thus demonstrating persistent pain behavior up to day 14 post-surgery. By contrast, the ipsilateral paws of Sham-operated rats showed paw withdrawal thresholds that were comparable to those in the contralateral paws of Sham rats and the contralateral paws of PSNL rats, with no noticeable changes.

### 2.2. Neuronal Activity in the Superficial Spinal Dorsal Horn

**Figure 2:**
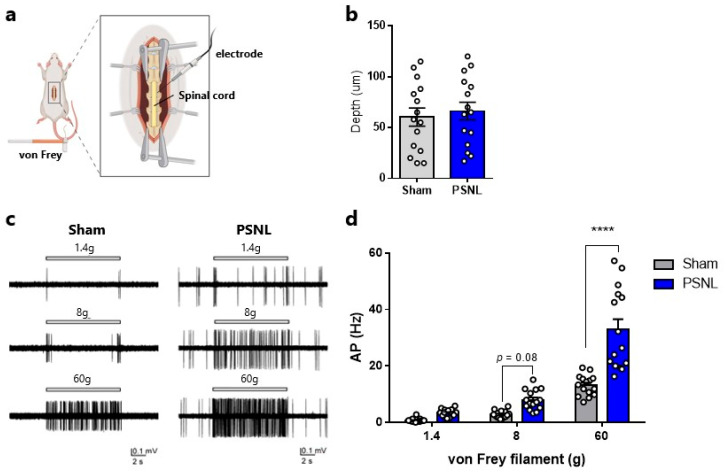
Stimulus-evoked neuronal activity in the superficial spinal dorsal horn of partial sciatic nerve ligation (PSNL) model rats. (**a**) Illustration demonstrating the *in vivo* extracellular recording procedure. (**b**) Recording depth. (**c**) Typical traces of neuronal firing by stimulation of the hind paw with 1.4, 8, and 60 g von Frey filaments in Sham and PSNL model rats. (**d**) Action potentials induced by the stimulation of the hind paw with a von Frey filament. Statistical analysis was performed using an unpaired *t*-test (**b**) or two-way analysis of variance with post hoc Šidák test (**d**). Data are shown as the mean ± standard error. (**b**,**d**) *n* = 15. **** *p* < 0.0001, vs. Sham. Created with BioRender.com (accessed on 18 December 2025).

Pain stimuli are transmitted via peripheral sensory neurons to superficial spinal dorsal horn neurons, and then to the brain. To determine neuronal activity changes in the superficial dorsal horn during von Frey stimulation, *in vivo* electrophysiological assessments were conducted (Figure 2a). Electrode depth did not significantly differ between the Sham and PSNL model rats (Figure 2b). Upon 60 g von Frey stimulation, significantly greater action potentials were observed in PSNL model rats than in Sham rats (Figure 2c,d). An increasing trend in action potentials was also observed with 8 g stimulation (Sham 2.81 ± 0.34 vs. PSNL 7.94 ± 0.91, *p* = 0.082, Figure 2c,d). Similarly, although apparent numerical increases were noted with 1.4 g stimulation, they did not reach significance (Sham 0.67 ± 0.18 vs. PSNL 3.31 ± 0.34, *p* = 0.583, Figure 2c,d).

### 2.3. Spontaneous Neuronal Activity in the Spinal Dorsal Horn

**Figure 3:**
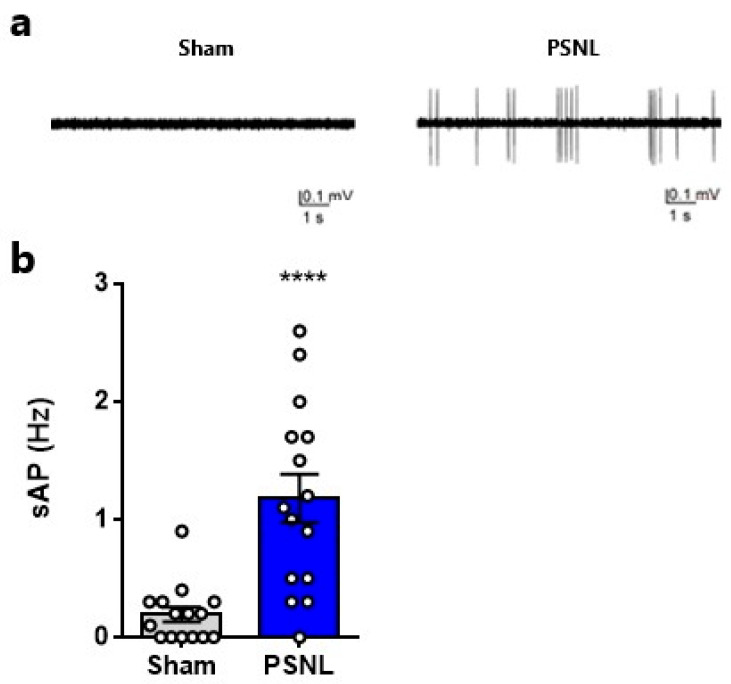
Spontaneous neuronal activity in the superficial spinal dorsal horn of partial sciatic nerve ligation (PSNL) model rats. (**a**) Typical traces of spontaneous neuronal firing in Sham and PSNL model rats. (**b**) Spontaneous action potentials in Sham and PSNL model rats. Statistical analysis was performed using an unpaired *t*-test (**b**). Data are shown as the mean ± standard error. (**b**) *n* = 15. **** *p* < 0.0001 vs. Sham.

**Figure 4:**
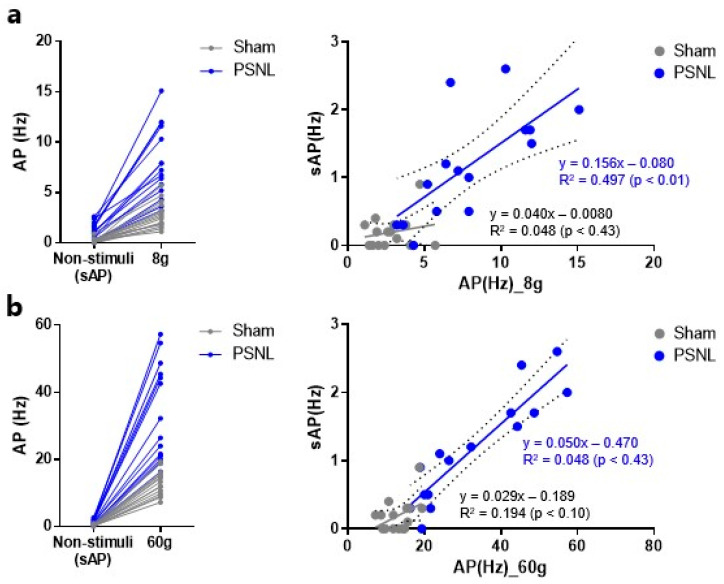
Correlation analysis between stimulus-evoked and spontaneous neuronal firing in the superficial spinal dorsal horn. (**a**) Correlation analysis between stimulus-evoked action potentials with 8 g von Frey filament and spontaneous action potentials in the superficial spinal dorsal horn. (**b**) Correlation analysis between stimulus-evoked action potentials with 60 g von Frey filament and spontaneous action potentials in the superficial spinal dorsal horn. Correlation analyses are shown separately for Sham-ipsi and PSNL-ipsi groups, and each dataset is labeled accordingly in the plots. Statistical analysis was performed using linear regression analysis (**a**,**b**).

To evaluate whether spontaneous activity in superficial spinal dorsal horn neurons is elevated in the absence of stimulation, *in vivo* electrophysiological evaluations of action potentials were performed without stimulation. There were significant increases in spontaneous action potentials in PSNL model rats (Figure 3a,b). To explore whether the increases in both spontaneous and evoked firing in spinal neurons within PSNL model rats originate from similar mechanisms at the cellular level, we analyzed the correlations between spontaneous neuronal firing and responsiveness to von Frey filament stimuli. There were significant correlations between spontaneous firing frequency and neuronal firing in response to 8 g von Frey stimulation (Figure 4a), and between spontaneous firing frequency and neuronal firing in response to 60 g von Frey stimulation (Figure 4b) in PSNL model rats but not in Sham rats.

### 2.4. Neuronal Activity in the DRG

**Figure 5:**
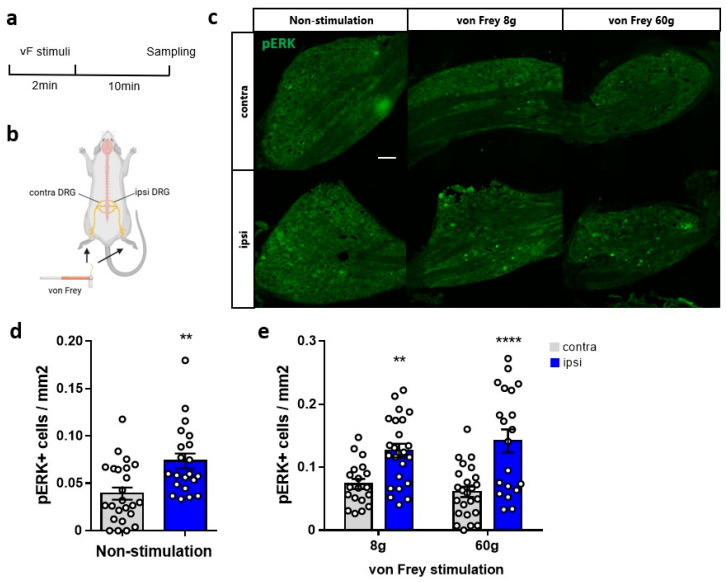
Neuronal activity in the dorsal root ganglion (DRG) of partial sciatic nerve ligation (PSNL) model rats. (**a**) Timeline of the process. (**b**) Schematic diagram highlighting the procedure. (**c**) Typical images of immunohistochemical staining of phosphorylated extracellular signal-regulated kinase (pERK; green) in the DRG of PSNL-contralateral (contra) and -ipsilateral (ipsi). Scale bars, 200 µm. (**d**) pERK-positive cell densities in the PSNL-contra and -ipsi DRG without any stimulation of the hind paw. (**e**) pERK-positive cell densities in the PSNL-contra and -ipsi DRG with 8 or 60 g von Frey stimulation to the hind paw. Statistical analysis was performed using an unpaired *t*-test (**d**) or two-way analysis of variance with post hoc Šidák test (**e**). Data are shown as the mean ± standard error. (**d**) Contra, *n* = 24; ipsi, *n* = 21 slices; ** *p* < 0.01 vs. contra. (**e**) 8 g contra, *n* = 19; ipsi, *n* = 24 slices; 60 g contra, *n* = 24; ipsi, *n* = 20 slices. ** *p* < 0.01, **** *p* < 0.0001 vs. contra. Created with BioRender.com (accessed on 18 December 2025).

To examine whether enhanced peripheral neuronal activity occurs during von Frey–induced withdrawal responses in the PSNL model, pERK immunostaining was performed in the DRG. pERK serves as a marker of peripheral neuronal activity; increases in its expression are correlated with neuronal activation [24]. This assessment involved the *in vivo* administration of von Frey filament stimuli followed by immediate DRG isolation to evaluate neuronal activity (Figure 5a,b). Considering the potential variability in fixation among rats, the contralateral paws of PSNL model rats (rather than the paws of Sham rats) were used as the control in the immunohistochemical study. In the absence of stimulation, the ipsilateral DRG of PSNL model rats exhibited significantly more pERK-positive neurons than the contralateral DRG (Figure 5c,d), which indicates heightened spontaneous peripheral nerve activity in the ipsilateral side. Additionally, we identified significant von Frey filament stimulus-dependent increases in pERK-positive neuron density in the ipsilateral DRG of PSNL models compared with the contralateral DRG with same stimulations (Figure 5c,e).

### 2.5. Correlations Between DRG pERK and Action Potentials of Superficial Spinal Dorsal Horn Neurons

**Figure 6:**
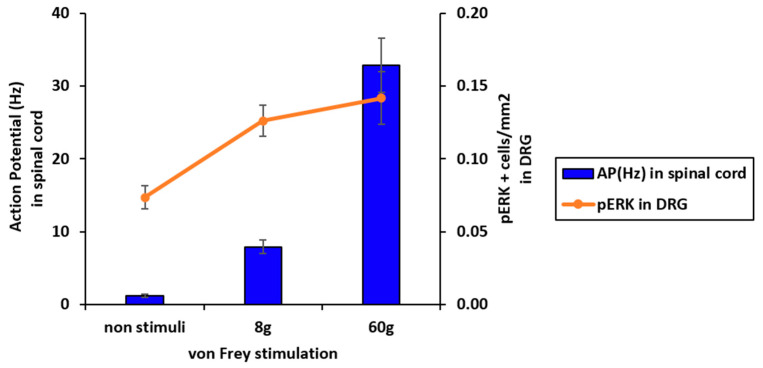
Comparison analysis between action potentials of superficial dorsal horn neurons and phosphorylated extracellular signal-regulated kinase (pERK)-positive cells in the dorsal root ganglion (DRG) in ipsi side of partial sciatic nerve ligation (PSNL) model rats. Bar graph indicates the action potentials of dorsal horn neurons in PSNL-ipsilateral (ipsi) measured using *in vivo* extracellular recording. Line graph indicates pERK-positive cell densities in the DRG of PSNL-ipsi measured using immunohistochemistry.

*In vivo* electrophysiological evaluations revealed increased spontaneous and stimulus-dependent activity in superficial spinal dorsal horn neurons (Figure 2 and Figure 3). Similarly, pERK immunostaining in the DRG indicated heightened spontaneous and stimulus-dependent neuronal activity (Figure 5). Together, these findings suggest the occurrence of potential peripheral–spinal dorsal horn circuitry changes that originate from heightened peripheral neuronal activity in PSNL model rats. To assess the correlations between changes in peripheral neuronal activity and superficial spinal dorsal horn neurons, pERK-positive neuron density in the DRG and action potential changes in spinal dorsal horn neurons with no stimulation and during 8 and 60 g von Frey filament stimulation were compared. Stimulus-dependent increases were observed across both parameters (Figure 6).

## 3. Discussion

Although the peripheral and central mechanisms of neuropathic pain have been extensively studied, most previous investigations have evaluated these domains in isolation. Comprehensive studies assessing both peripheral and spinal neuronal activity simultaneously—and exploring their functional relationships—remain scarce. Furthermore, few studies have used *in vivo* approaches that enable evaluations to occur under physiologically relevant conditions, particularly for both spontaneous and stimulus-evoked neuronal responses. In the present study, by combining *in vivo* electrophysiological recordings and immunohistochemical analysis, we demonstrated that PSNL model rats exhibit neuronal hyperactivity in both peripheral sensory neurons and spinal dorsal horn neurons. Our findings suggest that enhanced spontaneous firing and hypersensitivity to mechanical stimulation (e.g., von Frey) occur in both tissue types. Collectively, these results provide new insights into how peripheral nerve injury induces hyperresponsiveness along the pain transmission pathway.

The evaluation methods that were developed here are of considerable importance for both understanding the pathophysiology of neuropathic pain and developing effective therapeutic strategies. The ability to assess neuronal activity at both peripheral and spinal levels offers a valuable platform for profiling analgesic agents—particularly, for determining their sites of action. In parallel with these efforts, natural products and plant-derived bioactives have also gained attention as potential analgesic and neuroprotective candidates, partly due to their anti-inflammatory and antioxidant properties [25,26]. However, their actions are often multi-faceted, and the dominant site(s) of action are not always clearly established [27]. In this context, approaches that can functionally localize whether an intervention primarily modulates peripheral versus spinal neuronal activity are particularly valuable for mechanistic understanding and therapeutic development. Although peripherally acting agents such as Na_v_1.8 sodium channel inhibitors are believed to primarily target peripheral neurons, recent reports also suggest their possible spinal effects [28,29]. Thus, a simultaneous analysis at both levels is essential for mechanistic understanding as well as accurate drug evaluation. An additional advantage of our *in vivo* approach was the ability to apply the same stimuli as those used in behavioral assays, thereby allowing direct associations to be made between behavioral outcomes and the underlying neural mechanisms. Moreover, this method allows for advanced pharmacological profiling, especially when combined with newer techniques such as intrathecal drug delivery during electrophysiological assessment [30].

At the molecular level, peripheral nerve injury–induced hyperexcitability is mediated by coordinated changes in multiple ion-channel systems. In addition to altered voltage-gated sodium channel function, dysregulation of voltage-gated calcium channel pathways—particularly involving the auxiliary α2δ subunit targeted by gabapentinoids—has been implicated in neuropathic pain mechanisms and treatment [31]. This framework highlights why interventions acting on distinct channel systems may yield enhanced analgesic effects, and it provides a mechanistic context for interpreting multi-level changes in neuronal activity. In line with this concept, combination approaches that concurrently modulate sodium- and calcium-channel–related mechanisms have been reported to alleviate tactile allodynia in the partial sciatic nerve ligation model, supporting the rationale for dual-pathway modulation in injury-driven neuropathic pain [32].

From a pathophysiological perspective, our data suggest that in the PSNL model, in which peripheral nerve injury is the initiating event, neural hyperactivity likely originates in peripheral neurons and propagates centrally to the spinal cord. This concept supports the hypothesis that targeting peripheral hyperexcitability may be effective for treating neuropathic pain in injury-driven models such as the PSNL model. Importantly, we also detected increased spontaneous activity in both peripheral and spinal neurons. This may serve as a surrogate marker for spontaneous pain, which is a major clinical symptom that is difficult to assess in preclinical models. Although a direct correlation between spontaneous firing and spontaneous pain remains to be fully validated, our data provide a foundation for further investigations into translationally relevant endpoints. Our approach may also be extended to other pain models to identify the primary sites of neural sensitization. For example, in models involving central injury (such as cauda equina compression or spinal cord injury), it would be possible to determine whether hyperactivity is predominantly spinal or also involves peripheral neurons [33,34]. Similarly, in models of nociplastic pain such as fibromyalgia, our approach may help to clarify the anatomical origin of altered neural responsiveness, thereby informing targeted treatment development [35].

Nonetheless, the present study has several limitations. First, different techniques were used to assess neuronal activity in the DRG (pERK immunostaining) and spinal cord (electrophysiology). Although *in vivo* electrophysiological recordings from peripheral nerves are feasible, they involve mixed populations of fibers—C, Aδ, and Aβ—that are difficult to isolate and analyze individually. For this reason, we used pERK as a molecular marker, which has been validated as an activity-dependent signal that is upregulated by sensory stimuli such as a pinch, capsaicin, or heat, and is downregulated by local anesthetics such as lidocaine [24]. In our study, pERK expression was increased by von Frey stimulation relative to baseline in the ipsilateral DRG (Figure 6), thus supporting its validity as an activity marker. However, peripheral electrophysiological measurements and immunohistochemical evidence of spinal neuronal activation were not included, and incorporating these complementary assessments will be important in future work to further strengthen the peripheral–central framework. Second, we did not stratify DRG neurons by soma size. Although soma size is sometimes used as a rough correlate of fiber type, substantial overlap in soma size across A- and C-fiber populations limits the specificity of size-only classification [36]. Moreover, apparent soma size in thin sections varies with the sectioning plane. Future studies combining activity markers with subtype-specific markers (e.g., NF200, CGRP, IB4) and standardized morphometric workflows will be important to identify the contributing neuronal subpopulations. Third, neuronal activity was not tracked longitudinally across multiple postoperative stages. Behavioral outcomes were assessed longitudinally, whereas electrophysiology and immunohistochemistry were performed at a defined postoperative day 14. Time-resolved neuronal measurements will be valuable in future studies to better align neural activity dynamics with the behavioral trajectory. Finally, although spontaneous firing may reflect spontaneous pain, further validation is needed. Currently, no gold-standard behavioral assay exists for spontaneous pain. However, recent developments—such as sleep-based pain assessment—may enable correlation analyses once validated, and spontaneous neuronal activity may serve as a mechanistic surrogate in that context [37].

## 4. Materials and Methods

### 4.1. Animals

Male Sprague Dawley rats (5–7 weeks old) were obtained from CLEA Japan. Animals were maintained under standard conditions: a 12-h light/dark cycle (lights on at 8:00 a.m.), controlled ambient temperature, and free access to food and water. All procedures conformed to the regulations set by Japan’s Animal Welfare and Management Act and the Guidelines for the Care and Use of Laboratory Animals. The experimental protocol was reviewed and approved by the Institutional Animal Care and Use Committees at Shionogi & Co., Ltd., and the University of Toyama.

### 4.2. PSNL Model

The PSNL model was induced according to previously published procedures [38]. Rats were anesthetized with 4% isoflurane. The right thigh was shaved, and a skin incision was made. Underlying muscle tissue was parted to expose the sciatic nerve. A tight ligature was placed around the dorsal portion of the sciatic nerve using 4-0 nylon thread. On the opposite side (left) in PSNL model rats, the sciatic nerve was similarly exposed but was left intact to serve as a control (PSNL-contra). On the same side (right) in Sham model rats, the sciatic nerve was similarly exposed but was left intact to serve as a control (Sham-ipsi).

### 4.3. Behavioral Tests

Mechanical sensitivity was assessed by determining paw-withdrawal thresholds using the up–down method with von Frey filaments ranging from 0.4 to 60 g (North Coast Medical) [39]. Rats were placed onto a wire mesh floor inside a transparent inverted chamber. The plantar surface of each hind paw was stimulated at its central region using von Frey filaments. A withdrawal response was defined as a clear paw lift and/or retraction of the paw from the filament stimulation. The lowest force that elicited a withdrawal was recorded as the threshold.

### 4.4. In Vivo Extracellular Recordings

All *in vivo* extracellular recordings were performed on postoperative day 14. Extracellular recordings in live rats followed established protocols [40]. Urethane (1.2–1.5 g/kg, intraperitoneal) was used to achieve a stable anesthetic state without frequent top-ups. A laminectomy from L4–6 allowed access to the spinal cord. The animals were fixed in a stereotaxic frame, the dura was removed, and the arachnoid layer was opened to permit the insertion of a tungsten microelectrode into the dorsal horn. The exposed spinal cord was continuously perfused with Krebs solution (95% O_2_/5% CO_2_, flow rate 10–15 mL/minute) containing (in mM): 117 NaCl, 3.6 KCl, 1.2 MgCl_2_, 2.5 CaCl_2_, 11 glucose, 1.2 NaH_2_PO_4_, and 25 NaHCO_3_, with a temperature maintained at 37 ± 1 °C. Extracellular single-unit responses were recorded from neurons in laminae I and II of the dorsal horn at depths of 20–150 µm below the cord surface. Signals were amplified (EX1 amplifier, Dagan Corporation), digitized (Digidata 1400A; Molecular Devices), and acquired using Clampex v10.2. They were then analyzed using Clampfit v10.2. To assess stimulus-evoked responses, von Frey filaments (1.4, 8.0, and 60.0 g) were applied to the most sensitive sites in the receptive field on the hind paw for 10 s, and firing rates were recorded.

### 4.5. Immunohistochemistry

Immunohistochemical experiments were performed on postoperative day 14. Rats were anesthetized with urethane (1.5 g/kg, intraperitoneal), and von Frey stimuli were applied to the plantar surface every 10 s over a 2-min period. Animals were then perfused transcardially—first with cold phosphate-buffered saline (PBS), and then with formalin. The L4 DRG were harvested, fixed in formalin, and cryoprotected in 30% sucrose at 4 °C. DRG were then embedded in Tissue-Tek optimal cutting temperature compound (Sakura Finetech), cut into 10 µm sections using a cryostat, and mounted on glass slides. Sections were incubated overnight at room temperature in blocking solution (10% normal goat serum, 2% bovine serum albumin, and PBS with Tween 20) containing anti-pERK antibody (1:200, #4370S, Cell Signaling Technologies, Tokyo, Japan). After PBS washes, sections were exposed to Alexa Fluor 488–conjugated secondary antibody (anti-rabbit, 1:500; Thermo Fisher Scientific, Tokyo, Japan) for 1 h. Slides were then mounted with VECTASHIELD (Vector Laboratories, Burlingame, CA, USA), covered with coverslips, and observed using a BZ-X710 microscope (KEYENCE, Osaka, Japan). The pERK-positive cells in the DRG were quantified using ImageJ (version 1.5, National Institutes of Health, Bethesda, MD, USA).

### 4.6. Statistical Analysis

Data are presented as the mean ± standard error of the mean. For comparisons between two groups (non-paired), Student’s *t*-test was applied. Two-way analysis of variance followed by the Šidák post hoc test or Dunnett test was used for multi-group comparisons. Statistical analyses were conducted using GraphPad Prism version 6.0 (GraphPad Software).

## 5. Conclusions

Using *in vivo* electrophysiology and immunohistochemistry, the present study demonstrated that both peripheral and spinal neurons exhibit increased spontaneous and evoked activity in PSNL model rats. Collectively, these findings suggest that targeting peripheral neurons may be an effective therapeutic approach for peripheral neuropathic pain, such as that modeled by PSNL. Furthermore, the established methods offer a useful approach for investigating neuropathic pain and assessing the effects of potential analgesics. By applying this approach to other disease models, it is anticipated that optimal treatments will be devised for other conditions, thereby contributing to the development of new therapeutic drugs.

## Data Availability

The original contributions presented in this study are included in the article, and further inquiries can be directed to the corresponding author.

## Abbreviations

The following abbreviations are used in this manuscript:

DRG	dorsal root ganglion
PBS	phosphate-buffered saline
pERK	phosphorylated extracellular signal-regulated kinase
PSNL	partial sciatic nerve ligation
